# Integrating Genomic Information with Tumor-Immune Microenvironment in Triple-Negative Breast Cancer

**DOI:** 10.3390/ijerph192113901

**Published:** 2022-10-26

**Authors:** David Otohinoyi, Aditi Kuchi, Jiande Wu, Chindo Hicks

**Affiliations:** Department of Genetics, Bioinformatics and Genomics (Big) Program, School of Medicine, Louisiana State University Health Sciences Center, 533 Bolivar, New Orleans, LA 70112, USA

**Keywords:** triple-negative breast cancer, immune microenvironment, somatic mutations

## Abstract

Background: the development and progression of triple-negative breast cancer (TNBC) is driven by somatic driver mutations and the tumor-immune microenvironment. To date, data on somatic mutations has not been leveraged and integrated with information on the immune microenvironment to elucidate the possible oncogenic interactions and their potential effects on clinical outcomes. Here, we investigated possible oncogenic interactions between somatic mutations and the tumor-immune microenvironment, and their correlation with patient survival in TNBC. Methods: We performed analysis combining data on 7,875 somatic mutated genes with information on 1,751 immune-modulated genes, using gene-expression data as the intermediate phenotype, and correlated the resulting information with survival. We conducted functional analysis to identify immune-modulated molecular networks and signaling pathways enriched for somatic mutations likely to drive clinical outcomes. Results: We discovered differences in somatic mutation profiles between patients who died and those who survived, and a signature of somatic mutated immune-modulated genes transcriptionally associated with TNBC, predictive of survival. In addition, we discovered immune-modulated molecular networks and signaling pathways enriched for somatic mutations. Conclusions: The investigation revealed possible oncogenic interactions between somatic mutations and the tumor-immune microenvironment in TNBC, likely to affect clinical outcomes.

## 1. Introduction

Despite remarkable progress in patient management and screening, triple-negative breast cancer (TNBC) remains a major cause of breast cancer-related deaths [1,2]. TNBC is defined as breast cancer lacking expression of the estrogen receptor (ER), progesterone receptor (PR) and the human epidermal growth factor receptor (HER-2) [3]. Clinically, it is characterized by aggressive clinical behavior, high recurrence rates, poor prognosis and shorter survival time than patients affected by other subtypes of breast cancer [3,4]. Molecular and algorithmic classification have shown that TNBC is a highly heterogeneous disease, with a complex etiology [5,6,7]. Molecular-based classification has revealed six (6) subtypes of TNBC, including two basal-like (BL1 and BL2), immune modulatory (IM), mesenchymal (M), mesenchymal stem-like (MSL) and luminal androgen receptor (LAR) [6,7]. Currently, there are no effective Food and Drug Administration (FDA)-approved targeted therapies for the treatment of this aggressive, and often lethal, form of breast cancer. Due to the lack of well-defined molecular targets, surgery and cytotoxic chemotherapy using mainly anthracycline and taxane-based regimen remain the most effective therapeutic modalities [8,9]. Although chemotherapy treatment has shown improved clinical outcomes, appreciable instances of drug resistance still remain a challenge for standard chemotherapy [8,9]. Therefore, there is an urgent need for the discovery of prognostic markers and targets for the development of novel and more effective and less toxic therapeutics. 

The lack of targeted therapies and resistance to standard chemotherapy for TNBC has spurred interest in the development of cancer stem cell therapies [8,9] and immunotherapies [10,11]. Research on the use of cancer stem cells (CSCs) in the clinical management of breast cancer has been motivated by evidence from the published reports showing that cancer stem cells are substantially increased following conventional chemotherapy and neoadjuvant chemotherapy [8,9]. This phenomenon is of particular importance in TNBC, which has very limited therapeutic options and seems to be intrinsically enriched in CSCs, compared with other types of breast cancer [6,7,8,9]. Research on the use of immunotherapies in the clinical management of TNBC is motivated by the understanding that TNBC is the most immunogenic subtype of breast cancer, has the highest immune infiltrate, and its growth is influenced by the tumor-immune microenvironment [10,11,12]. There is a consensus in the oncology research community that evading antitumor immunity is a hallmark for the development and progression of TNBC cancer [10,11,12]. In addition, there is strong evidence that tumor-infiltrating lymphocytes in TNBC have a prognostic value, and are predictive of survival [10,11,12]. Therefore, the discovery of clinically actionable immunotherapeutic makers has the promise of improving the clinical management of TNBC and supplanting the use of cytotoxic chemotherapy. 

To date, the majority of immunotherapy studies have focused on elucidating the role of programmed cell death-1 (PD-1) receptor, programmed cell death-ligand 1 (PD-L1) pathway, and the checkpoint inhibitors [13,14,15]. PD-1/PD-L1 inhibitors have been used alone or in combination with platinum-based chemotherapy [13,14,15]. The PD-1 and PD-L1 pathway play a role in maintaining immunosuppression in the tumor microenvironment [13,14,15]. Consequently, blockade of the PD-1/PD-L1 axis has emerged as a promising therapeutic option in TNBC to enhance antitumor immunity [13,14,15]. However, despite the promise of immunotherapy, some patients do not respond to this therapy [13,14]. In fact, even those who do respond often develop resistance and relapse [13,14,15]. Therefore, there is a critical need to understand the interplay between the tumor-immune microenvironment and the tumor genome in TNBC. 

Traditionally, the development of prognostic markers has centered on the use of gene expression profiling [16,17,18,19,20]. Using transcription profiling, several clinically validated prognostic assays, among them PAM50, MammaPrint and Oncotype DX have been developed, and are being routinely used in the clinical management of TNBC [16,18,19]. However, the development and progression of TNBC originates from a more complex interplay between constellations of somatic driver mutations and the tumor microenvironment [13,14,15]. These complex arrays of interacting biological factors affect network states and signaling pathways which in turn drive disease aggressiveness and clinical outcomes. A more optimal approach to the discovery of clinically actionable biomarkers and potential therapeutic targets would be integrating data on somatic mutations with information on immune perturbation. 

Advances in next-generation sequencing technologies have enabled detailed analyses of TNBC genomes and the development of comprehensive catalogues of somatic mutations [21,22,23,24]. These analyses have provided valuable insights into the genomic basis and the molecular taxonomy of TNBC [21,22,23,24]. Recently, our group integrated information on somatic, epigenetic and gene expression variation in TNBC and mapped possible oncogenic interactions between these biological factors [25,26,27]. However, to date, there is little information in the published literature about the differences in somatic mutation burden between individuals who survive and those who die from TNBC. More importantly, there is a paucity of information on possible oncogenic interactions between the tumor genome and the tumor-immune microenvironment. The objective of this study was two-fold: (1) to determine whether there are differences in somatic mutations between individuals who survived and those who died from TNBC; and (2) to map possible oncogenic interactions between somatic mutations and the tumor-immune microenvironment. Our working hypotheses were that: (a) there are differences in somatic mutation profiles between individuals with a good prognosis who survive TNBC and individuals with a poor prognosis who succumb to the disease, (b) genes involved in immune perturbation (herein defined as tumor-immune microenvironment) are somatic mutated, and (c) that integrative analysis combing information on somatic mutations with the gene expression of immune-modulated genes would reveal possible oncogenic interactions between the tumor genome and the immune microenvironment and their potential effects on clinical outcome. We addressed these hypotheses using integrative and functional analysis combining somatic mutation with gene expression data from TCGA. For the purposes of this investigation, the tumor-immune microenvironment was captured and measured by genomic perturbations in all the immune-modulated genes regulating the immune repertoire, as described in the methods section and sources of information provided therein. 

## 2. Materials and Methods

### 2.1. Project Design and Execution Strategy

**Figure 1:**
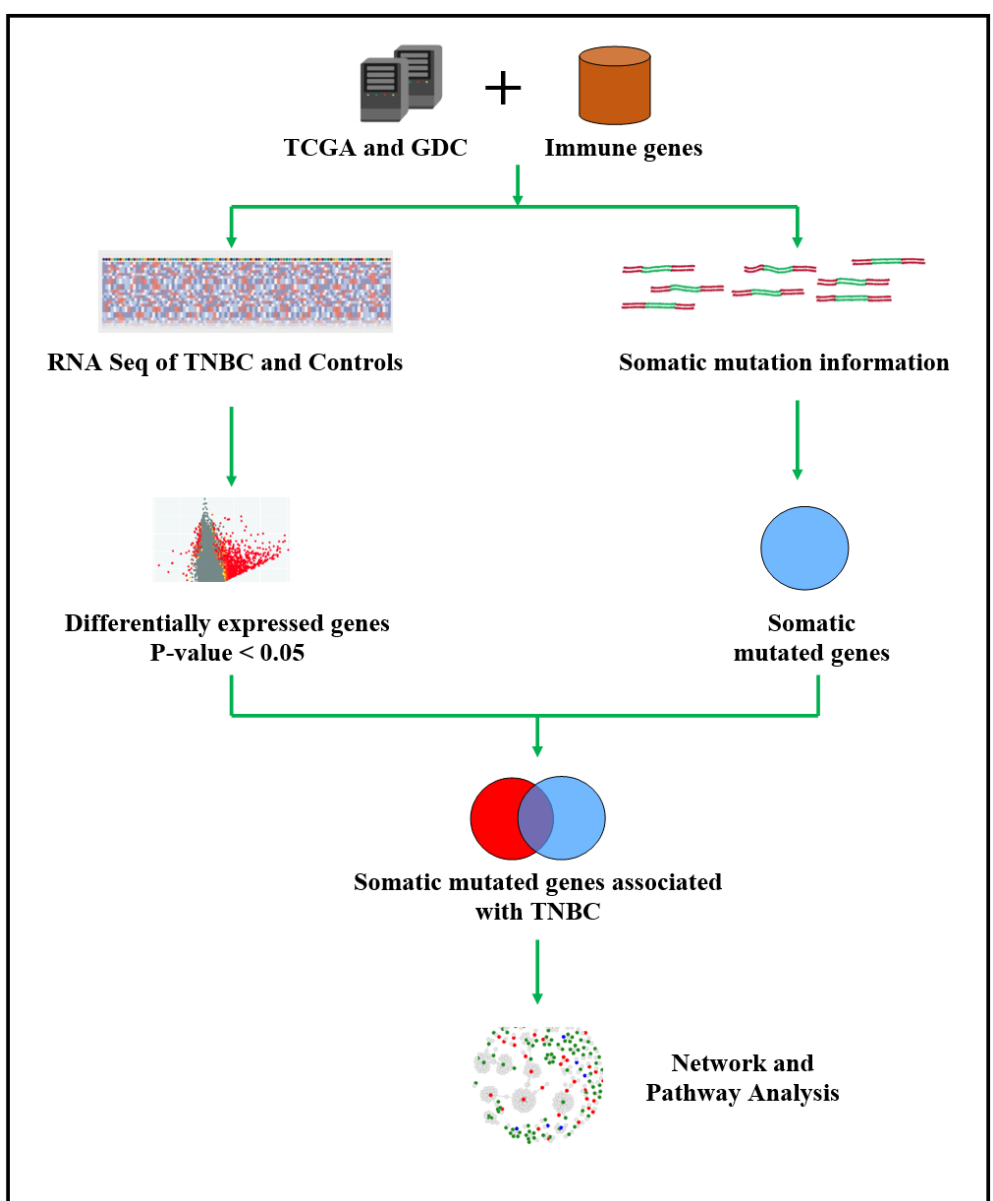
Overall project design, data processing and analysis workflow for the discovery of somatic mutated immune-modulated signatures of genes transcriptionally associated with TNBC, signatures predictive of clinical outcome; and immune-modulated gene regulatory networks and signaling pathways enriched for somatic mutations. In the figure, TCGA indicates The Cancer Genome Atlas, GDC indicates Genomics Data Commons, TNBC indicates triple-negative breast cancer, STN indicates solid tissue normal, DE indicates differentially expressed and DM indicates differentially mutated.

The development and progression of TNBC involves somatic driver mutations and the tumor-immune microenvironment [28]. Traditionally, analysis of somatic mutations and the tumor-immune microenvironment have been conducted as separate research endeavors. With the availability of information on somatic mutations and gene expression on genes perturbing the immune repertoire, we are now well-positioned to map possible oncogenic interactions between the tumor genome and the tumor-immune microenvironment and their potential impact on clinical outcomes. This investigation was conducted to address two longstanding issues. (1) To determine whether there are differences in somatic mutations between individuals diagnosed with TNBC with a good prognosis who survived and those with a poor prognosis who succumbed to the disease. (2) To map possible oncogenic interactions between somatic mutations and the tumor-immune microenvironment and their potential influence on outcomes, herein defined as deceased and alive. The overall project design and integrated analysis workflow is presented in Figure 1.

### 2.2. Sources of Genomics Data and Immune-Modulated Genes

We used publicly available gene expression and somatic mutation data linked with clinical information from TCGA on individuals diagnosed with TNBC and controls [21]. The data was downloaded from TCGA via the Genomics Data Commons (GDC) https://gdc.cancer.gov/ (accessed on 28 July 2021), a public resource of highly curated genomics data linked with clinical information, and an automated data portal supported with data download tools [29]. Using sample barcodes and identifiers, we linked molecular data with clinical information, and created a data matrix containing 228 samples distributed as 115 tumor samples and 113 control samples. Among the tumor samples, 97 individuals survived the disease (alive) and 18 individuals succumbed to the disease (died). The gene expression data set consisted of a total of 60,483 probes, generated using RNA-Seq. Gene expression, and somatic mutation data were generated on the same patient population, yielding a coherent data set suitable for integrative analysis. 

Somatic mutation data was generated using whole exome sequencing, consistent with TCGA protocol [21]. The mutation file contained 7,875 somatic mutated genes containing SNVs, inserts and deletions (indels). Using the sample IDs, we matched clinical with somatic mutation information to identify samples with information on both somatic and gene expression variation, as well as their outcome status. From this data processing step, we generated a data matrix containing both somatic mutations and gene expression on 115 TNBC tumor samples, and gene expression on 113 control samples. The data set was further stratified by clinical outcomes (survived, *n* = 97) and (deceased, *n* = 18). Molecular data was further linked with information on other clinical parameters, among them, tumor stage and presence (1) or absences (0) of metastasis. This information was used in downstream analysis for validation by correlating clinical information with molecular data on discovered biomarkers, to test their ability to predict clinical outcomes. All gene expression, somatic mutation and clinical information used in this investigation is publicly available and downloadable from the GDC https://gdc.cancer.gov/ (accessed on 28 July 2021) [29].

As noted in the introduction section of this report, the tumor-immune microenvironment was captured and measured by genomic perturbations in all the immune-modulated genes known to regulate the immune repertoire. The list of immune-modulated genes was constructed using the panels containing genes involved in regulating the immune repertoire in cancer used in immune experiments by Illumina and Qiagen [30,31]. Specifically, we used genes from Illumina (AmpliSeq) and Qiagen (QIAseq) immune response panels [30,31]. The genes were downloaded from Illumina’s Biospace and Qiagen databases [30,31]. The list was further ascertained and supplemented by immune response genes used in comprehensive analysis of immunologic portraits of TNBC [32,33]. It is worth noting that reports on the analysis of immunologic portraits of TNBC [32,33] did not include information on somatic mutations. In total, we evaluated a total of 1,751 immune-modulated genes. A complete list of all the immune-modulated genes used in this investigation is provided in Supplementary Materials in Table S7.

### 2.3. Bioinformatics and Statistical Data Analysis and Integration

The data processing and analysis workflow is shown in Figure 1. We processed the data and removed probe IDs with very low and or no expression values across all samples, from the gene expression data matrix. We mapped the probes onto the Ensemble database using BioMart, an Ensemble tool to identify the corresponding gene symbols or names [34]. The resulting gene expression data set with gene names was normalized using quantile normalization [35]. Using normalized data, we performed a multistage analysis. In the first stage, we compared gene expression levels between tumor samples and controls using a *t*-test implemented in Pomelo2 [36]. This unbiased approach was conducted to identify all genes significantly (*p* < 0.05) associated with TNBC, regardless of mutation and or immune modulation status. We used the false discovery rate (FDR) to correct for multiple hypothesis testing [37]. The resulting set of genes transcriptionally associated with TNBC were evaluated for the presence of somatic mutations across the patient population and within the two patient groups. We then compared the number and frequency of somatic mutations between individuals who survived (alive) and those who succumbed to the disease (deceased). We performed an independent *t*-test of unequal variances to compare mutation frequencies between the two patient populations. Use of unequal variances approach was necessitated by the unbalanced design nature of the study, that is unequal sample sizes between deceased and alive. The resulting information was correlated with tumor stage and metastatic status. A gene was considered differentially mutated if it had somatic mutations in only one patient group or if the somatic mutation found in one patient group was not found in the other patient group. Due to the sample size, we did not conduct analysis by subtype. 

In the second analysis stage, we evaluated all the 1,751 immune-modulated genes for their association with TNBC and for the presence of somatic mutations, by recapitulating the analysis conducted in the first stage. The genes were ranked on estimated *p*-values and FDR. We performed a quantitative assessment of the number and frequency of somatic mutations between individuals who survived (alive) and those who died, as described in the first analysis stage. 

### 2.4. Functional Analysis and Validation

We performed functional analysis using the Ingenuity Pathway Analysis (IPA) software platform [38] to discover immune-modulated molecular networks and signaling pathways enriched for somatic mutations. Under this approach, somatic mutated and immune-modulated genes uniquely associated with patients who succumbed to TNBC were mapped onto networks and canonical pathways, using IPA. We computed the Z-score to determine the likelihood of correctly assigning the genes to network state. The networks were ranked on Z-scores. We used Fisher’s exact *t*-test to determine the probability of correctly predicting the pathway onto which the gene maps. The pathways were ranked on log-*p*-values. We conducted Gene Ontology (GO) analysis implemented in IPA to categorize genes according to the cellular components, molecular functions and biological processes in which they are involved [39]. 

Finally, we conducted in silico validation of the discovered biomarkers to test their potential clinical utility. For this analysis, we evaluated somatic mutated immune-modulated genes transcriptionally associated with TNBC, using information on three clinically validated prognostic assays currently used in clinical management of TNBC [16,18,19]. The three assays were PAM50 [16], a 50-gene signature used to predict breast cancer survival and response to neoadjuvant chemotherapy, the main therapeutic modality for TNBC, MammaPrint [18], a 70-gene signature for predicting a breast cancer prognosis, and the Oncotype DX [19], a 21-gene assay that helps determine the risk for distant recurrence and whether chemotherapy is an appropriate course of treatment in patients with early-stage breast cancer. The three assays were developed using gene expression data, but have not been evaluated for the presence of somatic mutations and immune modulation in TNBC. 

## 3. Results

We performed integrative genomic analysis combining information on somatic mutations and immune modulation using gene expression data as the intermediate phenotype to determine whether there are differences in somatic mutation profiles between individuals who survive and those who die from TNBC, and to map possible oncogenic interactions between somatic mutations and the tumor-immune microenvironment. In this section and subsequent subsections, we present the results of the investigation. 

### 3.1. Discovery of Gene Expression and Somatic Mutated Signatures

**Figure 2:**
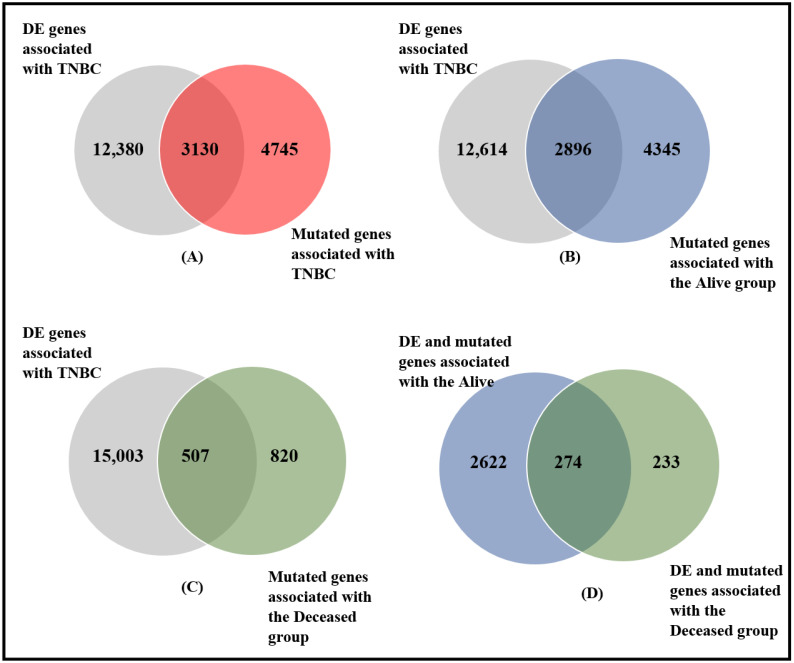
Distribution of somatic mutated and non-mutated genes significantly associated with TNBC: (**A**) genes altered in both the tumor genome and the transcriptome (see intersection), (**B**) genes with both somatic and expression alterations in the alive group (intersection), (**C**) genes with both somatic and expression in the deceased group (intersections), and (**D**) differentially mutated genes between alive and deceased patient groups transcriptionally associated with TNBC. In (**C**) genes in the intersections were associated with both groups. DE = differential expression.

The first objective of this investigation was to determine whether there are differences in somatic mutation profiles between individuals who survived and those who died from TNBC, and to discover somatic mutated gene signatures associated with each group of patients. To address this objective, we compared gene expression levels between tumor samples and controls, to discover a signature of genes transcriptionally associated with TNBC. The resulting set of genes were evaluated for the presence of somatic mutations across the entire patient population and compared between deceased and alive. The results of this investigation are summarized in Figure 2. 

Comparison of gene expression levels between tumor and control samples produced a signature of 15,510 significantly (*p* < 0.05) differentially expressed genes transcriptionally associated with TNBC (Figure 2A). Evaluating the 15,510 genes for the presence of somatic mutations produced a signature of 3130 somatic mutated genes transcriptionally associated with TNBC. Most of the mutations in the discovered genes were amino acid-altering. Among the somatic mutated genes transcriptionally associated with the disease which were discovered were the oncogenes *BRCA1*, *BRCA2* and *P53*, implicated in TNBC [23]. The most highly somatic mutated genes included *TP53*, *TTN*, *MUC4*, *USH2A*, *FAT3*, *MUC16*, *OBSCN*, and *SYNE1* with mutation counts of 76, 28, 13, 12, 12, 11, 10, and 10, respectively. A list of the top 30 most highly somatic mutated genes transcriptionally associated with TNBC, along with information on gene expression *p*-values and distribution of somatic mutation within each gene across tumor samples, is presented in Table 1. There was significant variation in the distribution of somatic mutations across the patient population and within the patient subpopulations under study. 

In addition to the discovery of somatic mutated genes transcriptionally associated with the disease, the investigation produced a signature of 12,380 genes transcriptionally associated with TNBC without somatic mutations (Figure 2A), and a signature of 4745 genes containing somatic mutations, but not transcriptionally associated with the disease (Figure 2A). Overall, the investigation revealed that integrative analysis of mutational and gene expression profiles provides a framework for identifying genetically altered genes that are transcriptionally associated with TNBC. A complete list of all the significantly (*p* < 0.05) differentially expressed genes transcriptionally associated with TNBC is presented in Supplementary Table S1A. A complete list of somatic mutated genes transcriptionally associated with TNBC along with their expression *p*-values is presented in Supplementary Table S2A. 

The discovery of genes transcriptionally associated with TNBC without somatic mutations and genes containing somatic mutations but not transcriptionally associated with the disease, was expected. This is because, while gene expression can be time specific, somatic alterations follow a clonal evolutionary pattern occurring at different time points during the development and progression of TNBC [23]. While we did not address the clonal evolutionally process of somatic mutations in this investigation, this has been reported in TNBC [23]. 

The results showing the signatures of somatic mutated genes transcriptionally associated with TNBC in patients with a good prognosis who survived, are presented in Figure 2B. The investigation produced 2896 somatic mutated genes transcriptionally associated with individuals who survived TNBC. In addition, the investigation produced 12,614 genes without somatic mutations transcriptionally associated with TNBC and 4345 somatic mutated genes not transcriptionally associated with the disease in individuals who survived (Figure 2B). 

A similar analysis focusing on individuals who died from TNBC produced a signature of 507 somatic mutated genes transcriptionally associated with the disease in individuals who died (Figure 2C). In addition, the analysis produced a signature of 15,003 genes without somatic mutations transcriptionally associated with the disease and 820 genes with somatic mutation but not transcriptionally associated with the disease (Figure 2C). 

### 3.2. Differences in Somatic Mutation Burden between Deceased and Alive

Having discovered somatic mutated genes transcriptionally associated with each patient subpopulation, we conducted an investigation to determine whether there are significant differences in the somatic mutation burden between individuals with a good prognosis who survived and individuals with a poorer prognosis who succumbed to TNBC, and to discover signatures of genes with somatic mutations unique to each patient group. To address this question, we compared the somatic mutation profiles of the two patient groups. The results are presented in Figure 2D. The investigation produced a signature of 2622 somatic mutated genes transcriptionally associated with TNBC for patients with a good prognosis who survived the disease, and a signature of 233 somatic mutated genes transcriptionally associated with the disease for patients with a poor prognosis who succumbed to the disease (Figure 2D). The results showing a list of the top most highly differentially mutated genes within the two patient groups are presented in Table 2. A complete list of all the differentially mutated genes transcriptionally associated with individuals who survived is presented in Supplementary Table S1B. A complete list of all the differentially mutated genes transcriptionally associated with individuals who died is presented in Supplementary Table S2B.

In addition, we discovered a list of 274 genes with somatic mutations in both patient groups (Figure 2D), see Supplementary Table S5. Interestingly, although 274 genes had somatic mutations in both patient groups, as shown in Supplementary Table S3, on an individual patient level, the mutation frequency number tended to be higher in those who died from the disease (only somatic mutations and clinical information on the deceased patients is presented in the Supplementary Table S3). The larger number of somatic mutations in patients with a good prognosis compared to those with a bad prognosis can be partially explained by the very small sample size in the deceaseddeceased patients subpopulation. 

To investigate whether there were significant differences in somatic mutation profiles for the 274 somatic mutated genes in both patient groups, we performed an independent *t*-test of unequal variances to compare mutation frequencies between the two patient populations. Somatic mutation information was correlated with clinical variables, tumor grade and metastatic state. The results are summarized in Table 3. The analysis produced more mutation occurrences per patient in the deceased patient group than the alive patient group, t (19) = 9.09, *p* < 0.05. Among the genes differentially mutated between the two patient populations, the top ten highly significantly mutated genes associated with the deceaseddeceased group included *TP53*, *ACE*, *LAMB4*, *ITGAX*, *LRP2*, *FBXW7*, *BRCA1*, *NSD1*, *NCOR1*, and *NLRC5*. For patients with a good prognosis who survived the disease, the average number of somatic mutations per patient was 6.63 ± 2.5, whereas the average number of somatic mutations per patient in the deceased was 16.78 ± 4.6 (Table 3). Correlating mutation profiles with stage and metastatic showed that most of the patients in the deceaseddeceased group had late-stage tumors and were in metastatic state (Table 3). A complete list of somatic mutated genes common to both alive and deceaseddeceased group, along with mutation frequencies used in the calculations of the results in Table 3, is presented in Supplementary Table S3. 

### 3.3. Discovery of Somatic Mutated Immune Modulated Gene Signatures and Pathways

The second objective of this investigation was to investigate whether immune-modulated genes are somatic mutated, and to map possible oncogenic interactions between somatic mutations and the tumor-immune microenvironment. To address this question, we tested the 1751 immune-modulated genes for their association with TNBC, using gene expression data. We then evaluated the immune-modulated genes transcriptionally associated with TNBC for the presence of somatic mutations across the two patient groups and within each patient group. 

**Figure 3:**
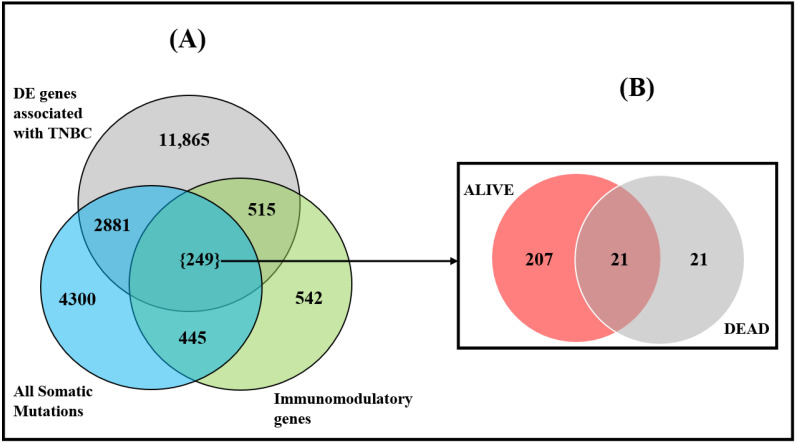
Distribution of somatic mutated and immune-modulated genes transcriptionally associated with TNBC (**A**), and stratified based on clinical outcome (**B**).

The results of the investigation are summarized in Figure 3. Out of the 1751 immune-modulated genes investigated, we discovered a signature of 764 immune-modulated genes transcriptionally associated with TNBC (Figure 3). Evaluation of these genes for the presence of somatic mutations produced a signature of 249 somatic mutated immune-modulated genes transcriptionally associated with the disease (Figure 3). In addition, the analysis produced a signature of 515 immune-modulated genes without somatic mutations transcriptionally associated with the disease, and a signature of 445 immune-modulated genes with somatic mutations but not transcriptionally associated with the disease (Figure 3A). 

Stratification of the patients by deceaseddeceased versus alive produced a signature of 207 somatic mutated immune-modulated genes associated with individuals who survived, 21 somatic mutated immune-modulated genes associated with individuals who died, and 21 somatic mutated immune-modulated genes transcriptionally associated with both patient groups (Figure 3B). A list of all the 42 somatic mutated immune-modulated genes associated with TNBC for individuals who died is shown in Table 4. A complete list of all immune-modulated genes transcriptionally associated with TNBC (with and without somatic mutations) is presented in Supplementary Table S4. Also presented in Supplementary Table S4 are lists of somatic mutated immune-modulated genes associated with each patient group and with both patient groups. In sum, the investigation revealed that a set of immune-modulated genes transcriptionally associated with TNBC are somatic mutated in the tumor genome. 

### 3.4. Oncogenic Interactions between Somatic and Immune Microenvironment

Having established that genes involved in the immune system are transcriptionally associated with the disease, and that a subset of these genes are somatic mutated, we performed functional analysis to map the possible oncogenic interactions between the two biological factors. Variant interpretation remains a challenge for precision oncology. Missense variants are particularly difficult to understand, as they change only a single amino acid in a protein sequence but can have large and varied effects on gene activity. Thus, because biological function arises through higher order interactions among protein-coding genes, we used a network- and pathway-based analysis approach. We sought to capture information about the potential of somatic mutations to perturb immune-modulated genes, their interaction networks and signaling pathways. Integrating information on immune modulation and somatic mutation data enabled us to gain insights into the broader biological context in which they operate, to drive adverse clinical outcomes. We performed network and pathway analyses using the 507 somatic mutated immune-modulated genes transcriptionally associated with TNBC in patients who died from TNBC. The rationale was that the discovery of immune-modulated network states and signaling pathways enriched for somatic mutations has the promise of revealing potential therapeutic targets. The networks were filtered, to keep only the most informative networks by removing spurious interactions (i.e. interactions with < 3 connections). 

**Figure 4:**
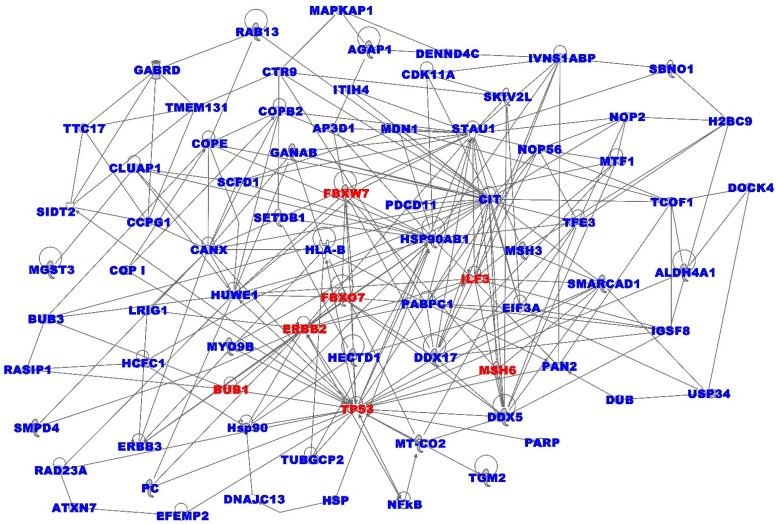
Molecular networks enriched for somatic mutations and immune-modulated genes in deceased patients diagnosed with TNBC. Genes in red fonts are somatic mutated immune-modulated genes associated with TNBC and genes in blue fonts are significantly DE and DM but not among the immune-modulated genes. Nodes represent genes, and vertices represent functional relationships.

The analysis produced 25 immune-modulated gene regulatory networks with scores ranging from 7 to 58, enriched with somatic mutations, which provided orthogonal functional information on genes classically used to prioritize variants in precision medicine. The results for the top seven most highly interconnected networks with the highest Z-scores, ranging from 40 to 58, are summarized in Figure 4. The analysis produced immune-modulated gene regulatory networks enriched for somatic mutations. The genes had multiple overlapping molecular functions. The networks included somatic mutated immune-modulated genes predicted to be involved in cancer, organismal injury and abnormalities, lipid metabolism, molecular transport, small molecule biochemistry, cell cycle, cellular compromise, DNA replication, recombination, and repair, hereditary disorder, cell morphology, cell death and survival. Among the significant immune-modulated gene regulatory networks associated with the clinical outcome in the deceased group were the prediction of cell-mediated immune response, immunological disease, inflammatory response, cellular function and maintenance, metabolic disease, and cell-to-cell signaling. A list of all the predicted networks and the somatic mutated genes mapping to them is presented in Supplementary Table S5. Overall, the investigation revealed the interplay between the somatic mutation and regulatory landscape in high-grade tumors with poor clinical outcomes. 

Pathway analysis produced 57 immune-modulated signaling pathways enriched for somatic mutations with -log *p*-values ranging from 1.0 to 4.6. Among the discovered signaling pathways are the antigen-presentation pathway, natural killer cell-signaling, phagosome formation, interleukin 4-signaling, p53 signaling, death receptor-signaling, and apoptosis signaling. Other significant pathways included pathways implicated in breast cancer, among them: actin cytoskeleton, the role of BRCA1 in DNA damage response, paxillin, UVA-induced MAPK, aryl hydrocarbon receptor, ILK, and the molecular mechanisms of cancer-signaling pathways. Additional pathways discovered were: hereditary breast cancer, the role of tissue factor in cancer, neuregulin, retinoic acid-mediated apoptosis, mismatch repair in eukaryotes, ERBB, PI3K/AKT, DNA damage-induced 14-3-3σ, GADD45, germ cell-sertoli cell junction, MYC mediated apoptosis, NGF, and HER-2 signaling pathways. A complete list of all the 57 signaling pathways perturbed by somatic mutations, along with associated genes and predicted -log *p*-values discovered in this investigation, are presented in Supplementary Table S6.

### 3.5. Validation and Potential Clinical and Translational Impact

To validate and investigate the prognostic value of the discovered somatic mutated immune-modulated genes transcriptionally associated with patients who died from TNBC, we used sets of genes from clinically validated prognostic assays PAM50, a 50-gene signature which helps predict breast cancer survival [16], MammaPrint, a 70-gene signature for predicting breast cancer prognosis [18], and Oncotype DX a genetic test for predicting risk of recurrence and resistance to chemotherapy treatment [19]. For this analysis we used the 507 somatic mutated immune-modulated genes transcriptionally associated with TNBC in individuals who died. Validation using the PAM50 assay revealed the genes *MYBL2*, *CENPF*, *ERBB2*; MammaPrint produced the genes *GPR180* and *ALDH4A1*, whereas Oncotype DX produced the gene *MYBL2*, all somatic mutated. The somatic mutated immune-modulated genes *CENPF* and *ERBB2* were found in the PAM50 assay. The discovery of somatic mutated immune-modulated genes transcriptionally associated with TNBC suggests that integrating information on somatic mutations and immune-modulated genes has the promise of a discovery of potential clinically actionable prognostic markers in TNBC. The discovery of fewer somatic mutated immune-modulated genes transcriptionally associated with TNBC present in the clinically validated assays evaluated was not surprising, because these assays were developed using only gene expression data. Gene expression can be time- and population-specific, whereas as mutations can follow a clonal evolutional pattern occurring at different time points during the course of the disease. Under such conditions, the observed outcome is expected. 

## 4. Discussion

The development and progression of TNBC is driven by somatic driver mutations and the tumor-immune microenvironment [28,40,41]. Traditionally, the analysis of somatic mutations and immune microenvironment have been conducted as separate research endeavors. Here, we combined the two pieces of information using gene expression data as the intermediate phenotype. We discovered a signature of somatic mutated immune-modulated genes. In addition, we discovered significant differences in somatic mutation profiles among somatic mutated immune-modulated genes transcriptionally associated with TNBC between individuals who survived and those who died from the disease. This suggest that there may be differences in the drivers of tumors and the tumor-immune microenvironment between individuals who died from the disease and those who survived. The involvement of the tumor-immune microenvironment in TNBC has been reported [28,40,41]. However, possible oncogenic interactions between somatic mutations and the tumor-immune microenvironment discovered here have not been reported. There are several potential clinical and translational aspects of these findings, which we articulate below. 

(*i) Differences in somatic mutation burden between deceased and alive individuals:* The observed differences in somatic mutation profiles between individuals who survived and individuals who died from the disease, and their correlation with tumor stage and metastatic status, suggests that TNBC patients may be amenable to mutation-based stratification. Although mutation-based classification was not attempted in this investigation, owing to the much smaller number of samples in the deceased group, multigene classification and prediction of survival as well as mutation-based classification in breast cancer have been reported [42,43,44,45], although those reports were not exclusively concerning TNBC. Our investigation examined all somatic mutations, some of which may not be drivers. While somatic driver mutations are the main contributors to TNBC [23], underlying passenger mutations may influence the somatic driver mutation landscape [46]. A recent study has shown that information on passenger mutations can be used to accurately classify human tumors [46]. 

(*ii*) *Discovery of somatic mutated immune-modulated gene signatures:* The discovery of signatures of somatic mutated immune-modulated genes associated with individuals who died from the disease and individuals who survived, suggests that the interplay between the tumor genome and the tumor-immune microenvironment in the two patient groups may be different. This has the promise of using combined therapies (chemotherapy and immunotherapy in the clinical management of TNBC [28,47,48,49]. Clinical reports have shown that Atezolizumab in combination with nab-paclitaxel, can be effectively used as a first-line treatment for patients with PD-L1-positive metastatic TNBC [28,47,48,49]. In addition, clinical trials investigating immune checkpoint blockade in early-stage TNBC have shown promise for the benefit of combining immune checkpoint blockade with neoadjuvant chemotherapy [47,48,49]. However, accumulating evidence from the published literature has shown that not all patients diagnosed with TNBC benefit from these treatments, as some patients develop resistance to these treatments [47,48,49]. The use of somatic mutation information in combination with these therapeutics could provide insights into the biological mechanisms underlying resistance to these therapeutics [47,48,49]. 

(*iii*) *Oncogenic interactions between somatic mutations and tumor-immune microenvironment*: The discovery of immune-modulated gene regulatory networks and signaling pathways enriched for somatic mutations was significant, given the emerging role of immunotherapy in TNBC [48]. Such pathways, if validated, could serve as targets for treatment and or development of more effective targeted therapeutics for TNBC. Integrative genomic analysis for the discovery of genetic determinants of immune phenotypes in breast cancer has been reported [50]. Our investigation focused on TNBC because there are currently no approved targeted therapies for this most aggressive, and often lethal, type of breast cancer. Because it is the most immunogenic cancer and has the highest immune infiltrate, it could be a candidate for immunotherapy [48,49]. Most notably, combining somatic mutations with immune-modulated genes could lead to understanding the molecular mechanisms underlying resistance to cytotoxic chemotherapy and the immunotherapeutic agents currently used in the treatment of the disease [47,48,49]. 

The discovery of somatic mutated and immune-modulated genes involved in the DNA repair machinery *BRCA1*, *BRCA2* and *P53*, was of particular interest because the majority of individuals diagnosed with TNBC carry BRCA1 and BRCA2 mutations [51]. Germline mutations in BRCA1 and BRCA2 predispose individuals to TNBC by impairing homologous recombination (HR) and causing genomic instability [51]. Importantly, DNA damage response (DDR)-associated therapies such as platinum-containing neoadjuvant chemotherapy have demonstrated potential efficacy against TNBC [51]. The discovery of genes involved in cell proliferation and DNA repair in this investigation was significant, because BL1 and BL2 subtypes of TNBC express high levels of genes involved in cell proliferation and DNA damage response [6,7]. The significance of this finding is that it suggests that patients with basal-like tumors would benefit from treatment with agents that preferentially target highly proliferative tumors such as DNA-damaging agents [7]. Moreover, information on DNA repair defects could be used to augment immune-based therapies in TNBC [52].

*(iv) Translational impact and clinical significance:* We did not investigate individual subtypes of TNBC because of limited sample size, or therapeutic modalities, because of limited sample size and lack of information on treatment in the data set used. However, a common feature of all subtypes of TNBC is that chemotherapy remains the standard and most effective therapeutic modality, but, unfortunately, patients frequently develop resistance [8]. The development of resistance to chemotherapy is multifaceted, and based on the elaborate interplay of the bulk tumor cells, cancer stem cells, the tumor-immune microenvironment, and drug efflux [8]. Here, we investigated the genomic alterations in bulk tumor cells and the tumor-immune microenvironment, and discovered multiple signaling pathways governing these interactions. We discovered immune-modulated signaling pathways enriched for somatic mutations implicated in TNBC, among them, the natural killer cell, phagosome formation, interleukin 4, p53, death receptor and apoptosis, the role of BRCA1 in DNA damage response, molecular mechanisms of cancer, hereditary breast cancer, ERBB, PI3K/AKT, MYC, and HER-2 signaling pathways implicated in TNBC [6,7], suggesting that such pathways could serve as therapeutic targets. 

The discovery of somatic mutated immune-modulated genes and pathways suggests that, if confirmed, these genes pathways could serve as prognostic biomarkers and therapeutic targets. Apart from the use of immunotherapy as an alternative to chemotherapy, there is growing interest in the use of cancer stem cell therapy, because accumulating data suggests that chemoresistant CSCs may be a dominant factor in TNBC relapse [8,53]. Because TNBC development and progression is driven by somatic driver mutations, and breast cancer stem cells are involved in TNBC formation, growth, invasiveness, chemotherapy resistance and tumor recurrence [8,9,50], the discovery of somatic mutated genes perturbing breast cancer stem cells could lead to a better understanding of the molecular mechanisms underlying resistance to chemotherapy, and to the development of more effective therapeutics that could overcome resistance. It is worth acknowledging that we did not study the link between cancer stem cells and immunotherapy, as that was beyond the scope of this investigation. However, there is evidence from the published literature supporting the link between cancer stem cells and immunologic markers with TNBC [8,9]. For example, a positive correlation between the expression of stem cell markers CD44 and ALDH1, and lower survival rates of TNBC patients has been reported [54,55]. Both CD44 and ALDH1 have been confirmed as immunologic markers for TNBC [56,57].

As noted in the introduction, TNBC is a heterogeneous disease. Among the six known subtypes, the LAR subtype is readily subclassified from other subtypes [6,7]. The LAR subtype of TNBC is characterized by the expression of the androgen receptor (AR), and has high levels of luminal cytokeratin expression [6,7]. Gene expression data analysis has shown that patients with the LAR subtype had higher relapse-free survival but no difference in distant metastasis-free survival, compared with all other TNBC subtypes, suggesting these patients have local relapse [6,7]. Whether this AR-driven subtype is arising from hormone replacement therapy (HRT) is not very clear, and merits further investigation [7]. However, there is growing interest in targeting the androgen receptor because of its link to the subtype, and also because of its involvement in several signaling pathways [6,7]. Accumulating evidence from the clinical management of TNBC suggests a role for anti-androgen therapies such as bicalutamide, enzalutamide and abiraterone [58]. These therapeutics offer an interesting chemo-free alternative for chemo-unresponsive patients [58,59]. While we did not investigate androgen therapy in this investigation, we discovered the PI3K/AKT, PI3K/mTOR, P53, ERBB and the DNA role of BRCA1 in DNA damage response signaling pathways, which display crosstalk with the AR signaling pathways. It has been shown that simultaneous targeting of the AR and the PI3K/mTOR signaling pathway may be clinically beneficial to LAR TNBC patients [6,7,58,59], as this combination has been shown to be synergistic in AR-dependent prostate cancer cells in clinical trials [60].However, the role of AR in TNBC is still not fully understood, especially in mesenchymal stem-like (MSL) TNBC cells. There is ample evidence from the literature demonstrating the tumorigenesis role of AR and the inhibitory effect of bicalutamide in AR-positive MSL TNBC, both in vitro and in vivo, suggesting that AR inhibition could be a potential therapeutic approach for AR-positive TNBC patients [61]. 

*Limitations:* This study provides valuable insights into the differences in somatic mutation profiles between individuals who survived and those who succumbed to TNBC, and the possible oncogenic interactions between the tumor genome and the tumor-immune microenvironment. However, limitations must be acknowledged. We did not control for multiple potential confounding factors in our analyses, such as copy number variants and DNA methylation status, which could also interact with both the tumor genomes and the tumor-immune microenvironment, and affect clinical outcomes. While such variables are important, including them was beyond the scope of this investigation. Not ithstanding this limitation, our previous studies on TNBC have shown that aberrant DNA methylation affects gene expression [25,26]. There is also ample evidence from the published literature that enduring epigenetic landmarks define the tumor microenvironment [62]. Indeed, the discovery of somatic mutated core cancer modules by integrating somatic mutation, copy number variation, and gene expression data, has been reported [63], but not concerning TNBC. Other notable limitations of this investigation are that our approach did not consider treatmment modalities such as chemotherapy, androgen, or immuno- and immune therapy in individual subtypes of TNBC and diverse populations. However, we have discussed the translational impact and clinical significance of our findings in relation to treatment options within the scope and limitations of the investigation. It is worth noting that, due to differences in genetic and ancestry background, and heterogeneity of the disease, both the immune repetoire and somatic mutation landscape may differ between subtypes and populations [64]. Likewise, therapeutic modalities such as chemotherapy, immunotherapy, cancer stem cells and androgen therapy may differ by subtype [7,48]. Due to a lack of information on treatment modalities in the data used, and because TCGA data has been generated using almost exclusively women of European ancestry, addressing the two issues was beyond the scope of this investigation. However, these weaknesses are readily acknowledged, and are worth pursuing in future lines of research. 

## 5. Conclusions

We discovered differences in somatic mutation profiles between individuals who survived and those who died from TNBC, suggesting that individuals diagnosed with TNBC may be amenable to mutation-based classification. The discovery of differentially mutated immune-modulated genes between survivors and deceased patients suggests that the biological mechanism driving the disease in the two patient subpopulations are likely to be different. The discovery of immune-modulated molecular networks and signaling pathways enriched for somatic mutations associated with individuals who died, confirms the interplay between somatic alterations and the tumor-immune microenvironment in driving the disease and potential clinical outcomes. Taken together, this investigation demonstrates that integrative analysis is a powerful approach for understanding the interplay between the tumor genome and the tumor-immune microenvironment. 

## Figures and Tables

**Table 1:** List of the top 30 somatic mutated genes highly significantly associated with TNBC.

Gene Symbol	Chrom Position	Ex-*p*-Value	SNVs	Deletions	Inserts	Total
CCDC74B	2q21.1	1.00 × 10^−6^	1	0	0	1
DCUN1D2	13q34	1.00 × 10^−6^	1	1	0	2
MAGEA4	Xq28	1.00 × 10^−6^	1	0	0	1
ANKRD34C	15q25.1	1.10 × 10^−6^	1	0	0	1
CAT	11p13	1.10 × 10^−6^	0	1	0	1
CCDC74A	2q21.1	1.10 × 10^−6^	3	0	0	3
EFR3B	2p23.3	1.10 × 10^−6^	4	0	0	4
FXYD3	19q13.12	1.10 × 10^−6^	1	0	0	1
GYG2	Xp22.33	1.10 × 10^−6^	4	0	0	4
IGLL5	22q11.22	1.10 × 10^−6^	1	0	0	1
LYST	1q42.3	1.10 × 10^−6^	6	0	0	6
MAPK8IP3	16p13.3	1.10 × 10^−6^	1	0	0	1
MT-CO2	Mitochondria	1.10 × 10^−6^	2	0	0	2
SMARCE1	17q21.2	1.10 × 10^−6^	1	0	0	1
SPECC1L	22q11.23	1.10 × 10^−6^	1	0	0	1
VWA2	10q25.3	1.10 × 10^−6^	1	0	0	1
C15orf39	15q24.2	1.20 × 10^−6^	5	0	1	6
DYNC1LI1	3p22.3	1.20 × 10^−6^	0	0	1	1
FLNB	3p14.3	1.20 × 10^−6^	1	0	0	1
GLYATL2	11q12.1	1.20 × 10^−6^	1	0	0	1
LINC00634	22q13.2	1.20 × 10^−6^	0	1	0	1
MT-ND4	Mitochondria	1.20 × 10^−6^	1	0	0	1
CNNM2	10q24.32	1.30 × 10^−6^	3	0	0	3
DIEXF	1q32.2	1.30 × 10^−6^	1	0	0	1
DOCK1	10q26.2	1.30 × 10^−6^	1	0	0	1
ERH	14q24.1	1.30 × 10^−6^	1	0	0	1
FREM2	13q13.3	1.30 × 10^−6^	4	0	0	4
LAMP3	3q27.1	1.30 × 10^−6^	1	1	0	2
NSD1	5q35.3	1.30 × 10^−6^	4	1	0	5
SLCO4C1	5q21.1	1.30 × 10^−6^	1	0	0	1

Chromosome positions were validated using the HUGO Gene Nomenclature Committee Database.

**Table 2:** Top highly differentially mutated genes transcriptionally associated with TNBC in either alive or deceased.

Gene Symbols	Chromosome Position	Mutation Frequency in the Alive Group	Mutation Frequency in the Deceased Group
DST	6p12.1	8	
RYR1	19q13.2	8	
CSMD2	1p35.1	7	
MUC5B	11p15.5	7	
MYO18B	22q12.1	8	
DYNC1H1	14q32.31	6	
ARID1B	6q25.3	6	
SACS	13q12.12	6	
CACNA1B	9q34.3	6	
RYR2	1q43	6	
ASPM	1q31.3	7	
MXRA5	Xp22.33	6	
CSMD3	8q23.3	7	
LRP1	12q13.3	6	
PREX1	2p14	5	
DSCAM	20q13.13	5	
SCN11A	21q22.2	5	
FHOD3	3p22.2	5	
DNAH5	18q12.2	5	
DIDO1	5p15.2	5	
HSPG2	20q13.33	5	
R3HCC1L	10q24.2		2
ZNF689	16p11.2		2
SYN2	3p25.2		2
C1orf167	1p36.22		2
PAQR8	6p12.2		2
NT5DC2	3p21.1		1
EYA1	8q13.3		1
LTK	15q15.1		1
SUGP2	19p13		1
CCDC39	3q26.33		1
CEACAM5	19q13.2		1
LZTS2	10q24.31		1
ARMC5	16p11.2		1
FAM161A	2p15		1
CPEB2	4p15.32		1
ITGB1	10p11.22		1
ZNF860	3p24.1		1
CANX	5q35.3		1
KRT8P12	3q25.33		1
HAUS3	4p16.3		1
SEL1L3	4p15.2		1

**Table 3:** Correlating information on differentially expressed somatic mutated genes common to both patient groups with clinical information.

Parameters	Alive Group (*n* = 97)	Deceased Group (*n* = 18)
Average number of mutations per patient across the 274 genes common to the outcomes (mean ± standard deviation)	6.63 ± 2.5	16.78 ± 4.6 *
Metastasis occurrence	0	2
Predominant tumor stage	Stage 1 & 2	Stage 3 & 4

* Indicates statistically significant difference from the Alive group.

**Table 4:** Somatic mutated immune-modulated genes associated with TNBC in the deceaseddeceased group.

Gene Symbols	Chromosome Position	Mutation Frequency in the Alive Group	Mutation Frequency in the Deceased Group
BMP5	12p13.33	0	1
BCL6	20q13.33	0	1
IFI35	12p13.31	0	1
HLA-DMA	3p21.31	0	1
NGF	14q22.1	0	1
EYA1	17q25.3	0	1
MAVS	15q26.1	0	1
MFGE8	8q21.11	0	1
FBXO7	19p13.2	0	1
ADA	4q31.3	0	1
FCER1A	8p12	0	1
IL21R	4q13.3	0	1
LTK	6p21.31	0	1
SFRP2	12p13.33	0	1
MGMT	12q13.3	0	1
PAK3	14q12	0	1
ALCAM	5p15.33	0	1
KNG1	5q31.1	0	1
CEACAM5	15q25.2	0	1
TSHR	7p13	0	1
KLRF1	19q13.11	0	1
TP53 *	17p13.1	64	13
ACE *	17q23.3	4	1
LAMB4 *	7q31.1	3	1
ITGAX *	16p11.2	3	1
LRP2 *	2q31.1	4	1
FBXW7 *	4q31.3	4	1
BRCA1 *	17q21.31	4	1
NSD1 *	5q35.3	2	2
NCOR1 *	17p12-p11.2	2	1
NLRC5 *	16q13	1	1
CENPF *	1q41	1	1
MSH6 *	2p16.3	2	1
ILF3*	19p13.2	1	1
CDH1 *	16q22.1	1	2
ERBB2 *	19p13.3	1	1
APC *	17q12	2	1
CAPN2 *	5q22.2	1	1
NFATC4 *	1q41	1	1
BUB1 *	14q12	1	1
HRAS *	2q13	1	1
ARHGEF1 *	19q13.2	2	1

* Genes that are common to both deceased and alive, but significantly mutated in the deceased group, *p* < 0.05.

## Data Availability

This study used publicly-available data on triple-negative breast cancer from The Cancer Genome Atlas (TCGA). Complete original data sets on RNA-Seq-generated gene expression data, Exome-Seq-generated somatic mutation data and clinical information used in this investigation are available at the TCGA https://www.cancer.gov/about-nci/organization/ccg/research/structural-genomics/tcga (accessed on 28 July 2021) and are downloadable via the Genomics Data Commons (GDC) at https://gdc.cancer.gov/ (accessed on 28 July 2021).

## Supplementary Materials

The following are available online at https://www.mdpi.com/article/10.3390/ijerph192113901/s1, Supplementary Table S1A—list of significantly differentially expressed genes between tumor and control samples associated with the TNBC. Supplementary Table S2A—complete list of somatic mutated genes significantly differentially expressed between tumor and control samples associated with TNBC. Supplementary Table S1B—lists of somatic mutated genes transcriptionally associated with individuals who survived. Supplementary Table S2B—lists of somatic mutated genes transcriptionally associated with individuals who died. Supplementary Table S3—list of 274 genes and mutation information for genes common to both patient groups used in calculation of differences in mutation frequencies between the two patient groups and clinical outcomes (list for deceased on the left and for alive on the right). Supplementary Table S4—lists of immunomodulatory and somatic mutated genes transcriptionally associated with TNBC, and stratified by deceased or alive. Supplementary Table S5—complete list of molecular networks containing somatic mutated immune-modulated genes transcriptionally associated with TNBC in the deceased. Supplementary Table S6—complete list of signaling pathways containing somatic mutated immune-modulated genes transcriptionally associated with TNBC in the deceased. Supplementary Table S7—comprehensive catalogue of 1751 immune-modulated genes used in the investigation.
